# Ketamine in Chronic Pain: A Review

**DOI:** 10.7759/cureus.53365

**Published:** 2024-02-01

**Authors:** Ana Faísco, Rita Dinis, Tânia Seixas, Luís Lopes

**Affiliations:** 1 Department of Anaesthesiology, Hospital Professor Doutor Fernando Fonseca, Amadora, PRT

**Keywords:** cancer pain, non-oncological pain, chronic postoperative pain, chronic pain, ketamine

## Abstract

Ketamine has been used in the treatment of several pain syndromes, particularly those with a relevant neuropathic component. Sub-anesthetic doses of ketamine produce a potent analgesic effect, due to its inhibition of N-methyl-D-aspartate receptors and enhancement of descending inhibitory pathways. Its short-term analgesic effect is well-documented perioperatively, with an associated reduction in postoperative chronic pain and opioid consumption. Despite some evidence regarding its long-term benefits, the number of clinical studies is still limited. In addition to its analgesic effects, ketamine also possesses an anti-depressive action, which might be useful in the treatment of chronic pain patients. Several side effects have been described, the psychomimetic ones being the most relevant due to their impact on treatment adhesion. At present, co-administration of ketamine and benzodiazepines or α2-agonists facilitates its clinical application. Despite current evidence and increasing use, further investigation is still needed regarding its long-term safety profile and clearer risk-benefit analysis.

## Introduction and background

Worldwide, the number of chronic pain patients is increasing. In Portugal, according to available data from 2012, 36% of adults suffer from some form of chronic pain, with about half of those reporting to be moderately or severely affected in their daily domestic or work activities [1].

The therapeutic approach to chronic pain should be guided by multimodal principles, making use of different categories of drugs, as well as psychotherapy techniques, physical rehabilitation practices, and more invasive but targeted interventions. Despite the breadth of knowledge and options available, chronic pain treatment still remains challenging, and its outcomes are frequently unsatisfactory [2].

Drugs, such as opioids, antidepressants, or anti-epileptics, are among the most frequently prescribed, but they are often associated with adverse effects, which can limit dosage and thus effectiveness [3]. As such, research into pharmaceutical alternatives and different drug combinations has remained important. Ketamine was introduced commercially in 1970 as a non-barbituric general anesthetic with a rapid onset and a particular mechanism of action, inducing a state of dissociative anesthesia with analgesia and preservation of the cardio-respiratory function. Its safety profile quickly garnered popularity, and its usefulness is not limited to the operating floor, but also in emergency rooms, prehospital, or intensive care.

Over the past few years, recognition of its analgesic properties in sub-anesthetic doses, the possibility of administration via several routes, the increasing number of patients with refractory chronic pain, and the opioid epidemic have brought renewed interest in its usage for the potential treatment of chronic pain and motivated the publication of a number of clinical trials, with promising results. Nasal administration produces rapid maximum plasma ketamine concentrations with relatively high bioavailability, and nasal sprays have been developed.

## Review

Historical considerations

Ketamine, a phencyclidine derivative, was first synthesized in 1962 by Calvin Lee Stevens, with the goal of being a less hallucinogenic and shorter-acting alternative to its parent compound [4]. Its mechanism of action was only uncovered 10 years after its clinical introduction when the glutaminergic system and related N-methyl-D-aspartate (NMDA) receptors were first described and their role in the functioning of the central nervous system (CNS) duly identified [5].

During the 1980s, its widespread usage decreased due to concerns regarding the well-known psychomimetic side effects. Despite this, the apparent effectiveness and usefulness in chronic pain settings, as well as neurologic and psychiatric conditions, have brought about renewed interest in its application.

Structural characteristics

Chemically, the ketamine molecule possesses a chiral structure consisting of an S(+) and R(- ) enantiomers. The S(+) enantiomer has twice the analgesic and anesthetic potency of the racemic mixture (equimolar mixture of both enantiomers), while the R(-) enantiomer is a more potent airway smooth muscle relaxant and has shown to provide longer and more effective antidepressant effect [6]. Despite only being available as a racemic mixture in Portugal, S(+) ketamine is commercialized in several other countries.

Mechanism of action

The primary mechanism of ketamine’s action is the non-competitive antagonism of the NMDA glutaminergic receptor in the brain and spinal cord. By interaction with these receptors, ketamine blocks calcium channels and leads to a subsequent reduction in the neurotransmission related to the excitatory amino acids glutamate and aspartate. Such antagonism translates clinically in its unique combination of effects: amnesia, an altered psychosensory state, and analgesia [7]. Ketamine’s action is dose-dependent, with a higher dosage producing anesthesia, and a lower one contributing only to analgesia. NMDA receptors have been described as playing an important role in pain transmission and modulation. They seem to contribute to such processes as central sensitization and wind-up phenomenon, two of the mechanisms seemingly responsible for the development of chronic pain [8]. In the presence of repetitive stimulation of C-fibers, NMDA receptors develop enhanced spontaneous activity. In theory, this cycle can be disrupted by ketamine, thus potentially preventing the progression of chronic pain [9].

Ketamine has also shown some affinity for other CNS receptors. Part of its analgesic properties may derive from μ, δ, e, and κ opioid receptor agonism [10], and its psychomimetic and sympathomimetic effects may result from interactions with monoaminergic receptors [11]. In addition to the well-established interaction with NMDA receptors, some evidence also suggests that ketamine may influence descending inhibitory pathways by activating monoaminergic descending inhibitory pathways at the supraspinal sites to produce anti-hypersensitivity effects [12]. Chronic pain patients often show deficient activation of such neural tracks [13].

Pharmacokinetics

After its absorption, ketamine quickly crosses the blood-brain barrier, providing a rapid onset of action. Peak plasma concentrations occur one minute after intravenous (IV) administration, five to 15 minutes after intramuscular (IM) injection, and 30 minutes after oral administration. It has high bioavailability (93%) after IV or IM administration, but when administered orally, higher doses are required due to first-pass metabolism, resulting in a significantly lower bioavailability (10-30%) [14]. Biotransformation takes place in the liver, with the most important pathway involving N- demethylation by cytochrome P450, producing nor-ketamine, an active metabolite with 20-30% of the potency of ketamine. The metabolites are then excreted in bile and urine after glucuronidation [15]. The distribution half-life is seven to 11 minutes, and the elimination half-life is two to three hours [16].

Pharmacodynamics and clinical application

At the CNS level, this drug causes depression of the normal corticothalamic association function, increasing the activity of the limbic system, thereby inducing a state of dissociative anesthesia characterized by profound analgesia and amnesia, even in an awake patient with maintained protective reflexes [17]. Regarding the cardiovascular system and mediated by the sympathetic nervous system, ketamine induces an increase in heart rate, cardiac output, and blood pressure. In contrast to other anesthetics, this preservation of cardiac function makes it an excellent option for hemodynamically unstable patients.

Ketamine also exhibits a potent bronchodilator effect and can be chosen in the treatment of refractory asthmatic status [18]. Historically, ketamine has been avoided in patients with neurological pathology due to the potential increase in intracranial pressure (ICP) caused by increased cerebral blood flow [19]. However, after administration of a 1, 3, or 5 mg/kg bolus to head-injured patients with increased ICP under propofol sedation, the drug reduced ICP (level III evidence) [19]. The same result was achieved in pediatric patients [20]. Thus, it has been suggested that ketamine does not increase ICP in sedated and mechanically ventilated patients with traumatic brain injury [21], and it may also be a safe drug in the treatment of refractory status epilepticus [22].

Other effects that have been studied are anti-tumor and anti-inflammatory [23]. The NMDA receptor block has been reported to inhibit several tumor-related actions. Evidence also shows that ketamine possesses antidepressant qualities, and a sub-anesthetic dose produces almost immediate antidepressant effects [24].

Ketamine in patients with chronic pain

Initially marketed as an anesthetic agent, ketamine is progressively gaining prominence in the treatment of perioperative acute pain and chronic pain, both oncological and non-oncological. Its use has been particularly important in neuropathic pain states, with validated administration routes including IV, IM, subcutaneous, epidural, intrathecal, intra-articular, intranasal, oral, and topical. Sadove et al. were the first to explore the analgesic properties of sub-anesthetic doses of ketamine [25]. Their double-blind clinical study in postoperative patients compared differences in pain scores between low doses of ketamine, meperidine, and placebo, suggesting the useful clinical application of ketamine in sub-dissociative doses as an analgesic. The discovery of NMDA receptors and their mechanism of action further reinforced ketamine's interest as a potent anti-hyperalgesic agent due to its effects on pain processing and neuroplasticity [26].

Ketamine has been studied among several conditions, including complex regional pain syndrome, fibromyalgia, chronic neuropathic pain, and phantom limb pain [27]. In addition to its interest in pain management, patients with chronic pain can benefit from its antidepressant effects, as many of these patients deal with depressive symptoms.

Ketamine in the Prevention of Chronic Postoperative Pain

Approximately 10-50% of patients suffer from persistent pain after surgical intervention [28]. Postoperative chronic pain is an often-neglected entity, with significant repercussions for both the patient and society.

Prolonged pain after amputations, thoracotomies, and mastectomies is usually caused by iatrogenic nerve injury. Due to its ability to block NMDA receptors and thereby reduce central sensitization, the use of ketamine has garnered interest in the multimodal approach to the prevention and treatment of postoperative chronic pain. More than just an analgesic action, its effectiveness lies in its modulating effect on central sensitization induced by surgical incision, tissue destruction, or opioid administration, demonstrating anti-allodynic, anti-hyperalgesic, and preventive action against opioid-induced hyperalgesia [29].

For this purpose, perioperative regimens are described with bolus doses of <1 mg/kg or continuous infusions of 0.1 mg/kg/h, up to a maximum of 700 mg in 24 hours via IV, epidural, or subcutaneous routes, and 0.5 mg/kg every six hours via oral and sublingual routes [30].

Ketamine in Non-oncological Pain

Ketamine appears to improve chronic pain associated with various conditions characterized by neuropathic pain, migraine, fibromyalgia, vascular pain, and short-term temporomandibular pain [31]. However, long-term analgesic effects are less well-described. Long-term effects have been studied primarily in complex regional pain syndrome (CRPS). Sigtermans et al. demonstrated that treating CRPS type 1 with a 100-hour infusion of S(+) ketamine, titrated to a dose of 20-30 mg/h, resulted in pain relief lasting up to three months after treatment [32]. Similarly, a therapeutic regimen with a daily four-hour infusion for 10 days in patients with CRPS achieved the same long-term effect [33]. Despite significant improvement in pain intensity, there was no improvement in functional levels, and many patients experienced ketamine’s more frequent adverse effects.

There is evidence that the duration of the analgesic effect is determined by the duration of the ketamine infusion. However, high-quality studies with well-defined outcomes are still lacking to establish the effective dose, infusion time, and optimal administration route [31], aiming to optimize the analgesic effect and minimize the occurrence of adverse effects.

Ketamine in Cancer Pain

The majority of studies found in the literature analyze the analgesic effects of ketamine on non-oncological chronic pain. In oncologic pain, when used, ketamine is often administered in conjunction with opioids. Ketamine seems to allow not only for a reduction in the required opioid dose, minimizing its side effects and improving treatment adherence, but also enhances the efficacy of opioids in pain treatment. The effect of ketamine on neuropathic pain is superior to that of opioids, thereby improving pain levels in cancer patients with a neuropathic pain component. Moreover, ketamine has its own analgesic effect and interacts synergistically with opioids and can prevent opioid-induced hyperalgesia [34]. Nevertheless, current evidence is insufficient to recommend the use of ketamine in the treatment of oncologic pain [34].

Side Effects and Limitations

Ketamine preserves cardiovascular and respiratory functions, making a lethal overdose unlikely. However, the safety profile in more prolonged use is still unclear. In the CNS, the most significant effects are psychotropic or psychedelic [35]. Despite being dose-dependent, these effects can be present even at the sub-anesthetic doses used in chronic pain. Both internal and external perceptions are affected, causing visual and auditory hallucinations, paranoid ideation, anxiety, heightened perception of sounds and colors, and dissociation in time and space. Other effects include dizziness, vertigo, nausea/vomiting, nystagmus, memory alterations, dysphagia, and motor function changes [36]. Generally, these effects cease rapidly after the cessation of ketamine administration. Complete prevention of these side effects is not possible, but coadministration of benzodiazepines or α2-agonist drugs (clonidine or dexmedetomidine) significantly reduces their intensity [37].

Ketamine is a potent sialogogue. The inhibitory effect on muscarinic receptors can explain the increase in bronchial secretion and mucus formation after ketamine [38]. Anosmia, angina, hypertension, injection site irritation hyperalgesia, and allodynia are also documented in the literature [39].

Ketamine induces psychological addiction in chronic users and is a substance of abuse, limiting its use outside the hospital environment. Chronic administration of high doses of ketamine is also associated with severe urological conditions, such as cystitis and bladder hyperplasia [40], and liver dysfunction has been described with an increase in serum liver enzyme levels [41].

## Conclusions

The primary mechanism of action of ketamine is unique among anesthetic and analgesic drugs. Its psychosomatic effects limit its use, but therapeutic regimens with sub-anesthetic doses and coadministration of benzodiazepines or α2-agonists have increased its application in chronic pain treatment in recent years.

There is evidence that ketamine treatment for chronic pain, particularly pain with a neuropathic component, may provide prolonged analgesic relief. However, this evidence comes from a still limited number of studies. Therefore, conducting more high-quality controlled clinical trials is imperative to demonstrate the analgesic effects of ketamine with an acceptable risk-benefit ratio. As such, ketamine is not recommended as a first-line drug in the treatment of chronic pain but should remain a potential choice in the multimodal approach to chronic pain syndromes, especially those refractory to standard treatment.
